# Realist evaluation for programs and services in the health area: an integrative review of the theoretical and methodological literature

**DOI:** 10.1590/1518-8345.3933.3255

**Published:** 2020-10-19

**Authors:** Jeane Roza Quintans, Tatiana Yonekura, Carla Andrea Trapé, Cassia Baldini Soares

**Affiliations:** 1 Universidade de São Paulo, Escola de Enfermagem, São Paulo, SP, Brazil.; 2 Hospital do Coração, Laboratório de Implementação do Conhecimento em Saúde da Associação Beneficente Síria, São Paulo, SP, Brazil.

**Keywords:** Health Evaluation, Measurements, Methods and Theories, Stakeholder Participation, Public Policy, Public Health, Health Plan Implementation, Avaliação em Saúde, Medidas, Métodos e Teorias, Participação dos Interessados, Política Pública, Saúde Pública, Implementação de Plano de Saúde, Evaluación en Salud, Mediciones, Métodos y Teorías, Participación de los Interesados, Política Pública, Salud Pública, Implementación de Plan de Salud

## Abstract

**Objective:**

to identify and analyze the concepts of the realist evaluation and the methodologies recommended for its development in the health area.

**Method:**

an integrative review, which included theoretical and methodological studies published in the following databases: COCHRANE Library, EVIPNet, Health Systems Evidence, LILACS, PDQ-Evidence, PubMed, Rx for Change, and SciELO, in addition to *Teses-CAPES* and Google Scholar, for the gray literature. The *mediation* category underlay the analysis.

**Results:**

19 references were included, published between 1997 and 2018. It is an innovative proposal to direct the process of evaluating health programs, interventions, and/or policies, with the democratic participation of the parties involved, such as users, workers, managers and researchers; it proposes to elaborate theories about what works, for whom, in what context, and how. The *mediation* category indicated the need for these theories not to be restricted to the micro-context, but to incorporate the elements of the social macro-structure to which they are connected.

**Conclusion:**

It is indicated that the realist evaluation is to be conducted in 21 stages. It takes into account qualitative and procedural methods, which makes it powerful for understanding human and social relationships in the context analyzed. Theories that come from evaluating the functioning of the programs analyzed have greater explanatory chances if they are built by reference to the social totality.

## Abstract (pt)

**Objetivo:**

identificar e analisar os conceitos de avaliação realista e as metodologias recomendadas para o seu desenvolvimento na área da saúde.

**Método:**

revisão integrativa, que incluiu estudos teórico-metodológicos publicados nas seguintes bases de dados: COCHRANE *Library*, EVIPNet, *Health Systems Evidence*, LILACS, PDQ-*Evidence*, PubMed, *Rx for Change*, SciELO, além de Teses-CAPES e Google Acadêmico, para a literatura cinza. A categoria *mediação* fundamentou a análise.

**Resultados:**

foram incluídas 19 referências, publicadas entre 1997 e 2018. Trata-se de proposta inovadora, para direcionar o processo de avaliação de programas, intervenções e/ou políticas de saúde, com a participação democrática das partes envolvidas, como usuários, trabalhadores, gestores e pesquisadores; propõe-se a elaborar teorias sobre o que funciona, para quem, em qual contexto e como. A categoria *mediação* indicou necessidade de que essas teorias não se restrinjam ao microcontexto, mas incorporem os elementos da macroestrutura social aos quais estão conectados.

**Conclusão:**

indica-se que a avaliação realista seja feita em 21 etapas. Ela toma em consideração métodos qualitativos e processuais, o que a torna potente para compreender as relações humanas e sociais no contexto analisado. As teorias que advêm da avaliação sobre o funcionamento dos programas analisados têm maiores chances explicativas se forem construídas por referência à totalidade social.

## Abstract (es)

**Objetivo:**

identificar y analizar conceptos de evaluación realista y metodologías recomendadas para su desarrollo en el área de la salud.

**Método:**

revisión integradora, que incluyó estudios teórico-metodológicos publicados en las siguientes bases de datos: COCHRANE Library, EVIPNet, *Health Systems Evidence*, LILACS, PDQ-*Evidence*, PubMed, *Rx for Change*, y SciELO, además de la base de datos de Tesis de CAPES y Google Académico, para la literatura gris. La categoría de mediación justificó el análisis.

**Resultados:**

se incluyeron 19 referencias, publicadas entre 1997 y 2018. Se trata de una propuesta innovadora para orientar el proceso de evaluación de programas, intervenciones y/o políticas de salud, con la participación democrática de las partes implicadas como usuarios, trabajadores, gestores e investigadores; se propone elaborar teorías sobre lo que funciona, para quién, en qué contexto y cómo. La categoría de mediación indicó la necesidad de que estas teorías no se limiten al microtexto, sino que incorporen los elementos de la macroestructura social a los que están conectadas.

**Conclusión:**

se indica que la evaluación realista se realice en 21 etapas. Tiene en cuenta métodos cualitativos y de procedimiento, lo que la capacita para comprender las relaciones humanas y sociales en el contexto analizado. Las teorías que provienen de la evaluación sobre el funcionamiento de los programas analizados tienen mayores posibilidades explicativas si se construyen con referencia a la totalidad social.

## Introduction

The evaluation of complex health interventions, as is the case with the public health policies and programs, is considered a challenge, especially given the assumption that it must organically monitor the creation and implementation of these interventions^(^ 1 ^)^.

The World Health Organization stresses the importance of evaluations based on the human rights principles and advocates the involvement of the so-called stakeholders (parties who are interested in the changes promoted by the programs and policies), including beneficiaries, as they can contribute to a better understanding of the processes that bring about changes in a given reality^(^ 2 ^)^.

Complex interventions are often informed by elements of experience and are dependent on the resources of those who make health decisions. On the other hand, it is known that assumptions about the success of this type of intervention must be better understood through evaluation processes, since they occur concretely within the scope of social relationships, allowing to ascertain the plausibility of the intervention and assist the evaluators in deciding what should be prioritized^(^ 3 ^)^.

Procedural monitoring, with access by the team of evaluators to intermediate outcomes, is considered essential in the evaluation of complex health programs^(^ 4 ^)^.

The evaluations indicated for complex interventions involve stages that range from the identification of health needs to the design and implementation of the programs and policies. In addition, it is necessary that the evaluation process takes into account the discussion of the political priorities and considers collecting data at diverse moments, to capture changes over time^(^ 3 ^)^.

The traditional models for assessing public programs and policies do not have such attributes. Coming from predominantly positivist frameworks, they focus on the interests of the organizations and institutions, the logic of human resources, and the cost-benefit ratio. In this perspective, the structure is vertical, with the evaluator playing a central role and being generally external to the context of the evaluation^(^ 5 ^)^.

The realist evaluation aims to overcome this traditional approach. In this sense, it involves qualitative and quantitative components, based on theory, to promote the implementation of policies and programs in specific contexts^(^ 6 ^)^. The realistic review, which shares the same theoretical perspective with the realist evaluation, argues that the best evidence must come from a theoretically oriented and locally situated process^(^ 7 ^)^.

A literature review mapped the concepts of the realist evaluation as applied to health systems research, based on primary studies that used this evaluation methodology. The authors recommend greater clarity regarding the definitions of mechanisms and context, two elements considered structural in the realist evaluation, in addition to the outcome^(^ 8 ^)^.

Thus, this review aims at identifying and analyzing the concepts of the realist evaluation and the recommended methodology for its implementation in the health area.

## Method

The question in this review is the following: What are the concepts and stages of the realist evaluation used in the health area? It was formulated using the PICo strategy (P: Population; I: Phenomenon of Interest and Co: Context), with the elements delimited as follows: P - Without delimiting the population; I - Concepts and stages of the realist evaluation; Co: Health area.

The integrative review (IR) was chosen because it constitutes an appropriate methodology to contribute to the synthesis of the review of theories and methodologies^(^ 9 ^)^, thus stimulating the exposure and understanding of theoretical and methodological frameworks about certain phenomena of reality. The development stages can be systematized in eight steps, according to the literature in the area: (1) Creating a group to conduct the IR; (2) Preparing the introduction; (3) Formulating the question and the objective; (4) Describing the methodology; (5) Analyzing and interpreting data; (6) Presenting outcomes; (7) Interpreting and discussing outcomes; and (8) Disclosing outcomes^(^ 10 ^)^.

In this sense, once the first phases that demanded meetings of the responsible group were overcome, in stage 4 only theoretical and methodological studies, both published and of the gray literature, were included, which analyzed and/or proposed theories and/or methodologies of the realist evaluation. The search was carried out in August 2019 and included references published in Portuguese, English, and Spanish, in any year, and indexed in the databases until July 2019.

To search for scientific and gray literature, the expression “Realist evaluation” in English or Portuguese was used in the following databases: COCHRANE Library, EVIPNet, Health Systems Evidence, LILACS, PDQ-Evidence, PubMed, Rx for change, and SciELO, in addition to *Teses-CAPES* and Google Scholar. The expression “Realist evaluation” was chosen as it was the most sensitive search strategy, enabling the mapping of the largest number of potential references on the theme. On the other hand, it guaranteed sufficient specificity. This expression is not a MeSH term, as it is a relatively new term. Manual searches and among the references of the references included were also carried out.

After identifying the studies, the references were selected by title and abstract, full reading, inclusion/exclusion, and data extraction by the team of four reviewers, with at least two of them working independently. Data extraction was performed using an instrument composed of the following items: (1) Title; (2) Year; (3) Authors; (4) Country of origin of the lead author; (5) Area of knowledge of the first author; (6) Objective of the study; (7) Definition and purposes of the realist evaluation; (8) Theoretical reference framework; (9) Stages of development of the realistic review; and (10) Summary of the text selected.

The evaluation of the methodological quality of the studies included was not carried out, as the object of study was of the theoretical type, and there was no inclusion of empirical studies.

From the point of view of collective health, the theoretical approach of historical and dialectical materialism was adopted in this study^(^ 11 ^)^, with the essential conceptual elements of the realist evaluation being analyzed according to the mediation category.

The authors of this article declare that they have no conflict of interest.

## Results

428 references were identified from data sources, manual searches, and references of the references, as shown in Figure 1.

**Figure 1:** Data source, search strategies, and references identified and selected by title and abstract. São Paulo, Brazil, 2019

Database	Search strategy	References identified
PubMed	"realist evaluation"[Title/Abstract]	278
LILACS	realist [Palavras] and evaluation [Palavras]	5
Cochrane	"Realist evaluation" in Title, Abstract, Keywords in Trials'	36
EVIPNet	ti:(Realist evaluation) OR mh:(Realist evaluation)	0
Health Systems Evidence	"Realist evaluation"	3
Rx for change	"Realist evaluation"	0
PDQ Evidence	"Realist evaluation"	0
SciELO	realist [Abstract] and evaluation [Abstract]	2
*Teses-CAPES*	"avaliação realista"	5
Google Scholar	"Realist evaluation"	99
**Total**		**428**

**Figure 2:**
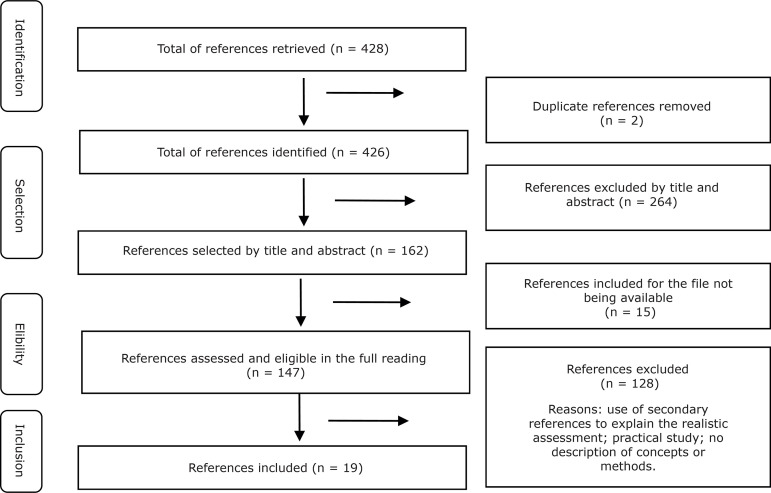
Flowchart of the article selection process. São Paulo, Brazil, 2019^(^ 12 ^)^

After excluding duplicate and unavailable publications and selection by title and abstract, 47 references were analyzed in full. In total, 19 references were included, as shown in Figure 2.

It should be noted that the studies were published between 1997 and 2018, with nine (47%) starting from 2011. The studies were published by authors from the United Kingdom, Australia, Canada, Sweden, United States, and Singapore, in the following areas of knowledge of the first author: Primary Care, evaluation, Education, Nursing, Philosophy, Business, Rehabilitation, Technology Development, Sociology and Social Policy, and Social Research. The figure below presents the integrative synthesis of the definitions and purposes of the realist evaluation:

Figure 3 describes the main elements of data extraction from the 19 studies included.

**Figure 3:** Author(s), year of publication, country of origin of the lead author, area of knowledge of the first author, and definitions and purpose of the realistic review. São Paulo, Brazil, 2019

N	Author(s)/Year/Country of origin of the lead author/Knowledge area of the first author	Definitions and purposes of the realist evaluation
1	Pawson, Tilley (1997) United Kingdom Sociology and Social Politics^(^ 6 ^)^	New evaluation paradigm, based on realism, which requires the use of different methods and data analysis that favor the explanation of the regularity and theory that underlies the logic of operation of the iteration of the contexts, mechanisms, and outcomes of the studied reality.
2	Henry, Rog (1998) Did not describe other data^(^ 13 ^)^	It develops a contextual understanding which reveals the mechanisms that generate different outcomes. It seeks to understand what types of evaluations are useful to which audiences, under which political conditions. The realist evaluation can be applied using the outcomes directly in making decisions about programs; using the outcomes to influence the way a program or its effects are seen; or using the outcomes to justify the decision about the programs.
3	Mark, Henry, Julnes (1998) Did not describe other data^(^ 14 ^)^	The emerging realist evaluation estimates the effects of programs and assesses activities, such as which social values are served by certain programs. Thus, it seeks to identify the mechanisms underlying the effects of the program, the conditions under which these mechanisms operate, and the types of individuals for whom they operate.
4	Tilley (2000) Did not describe other data^(^ 15 ^)^	It provides information to support public policy decision making and implementation. The realist evaluation question comprises the following: What works, for whom, and in what circumstances? Based on an understanding of how the measures will produce varying impacts on different circumstances, it is believed that the policy maker will be better prepared to decide which policies to implement under which conditions.
5	Kazi, Rostila (2002) England Evaluation studies^(^ 16 ^)^	It incorporates the main mechanisms, contexts, and components of the programs in the evaluation process. One of the main contributions of the realist evaluation paradigm is the concept of inserting the evaluator in the organizational process/structure. The realist evaluation considers the influence of the social relationships and organizational structures that make up the open system on the program's outcomes. As it is an open system, it is necessary to identify some regularities, that is, characteristics, factors, and mechanisms that lead to better or worse outcomes, and to identify the conditions under which the causal mechanisms would be activated to produce the outcomes.
6	Pawson, Tilley (2004) Did not describe other data^(^ 17 ^)^	An evaluation guided by theory, with an explanatory search. In the evaluation process, the theories are tested with the purpose of refining them. The authors highlighted the need to understand the nature of the programs and how they work, considering the following: 1) The nature of the programs and how they work; 2) Basic concepts for understanding programs that involve mechanism, context, and results. 3) Strategies and methods of the realist evaluation; 4) Presentation and use of the outcomes of the realist evaluation by the policy maker, in order to understand the issues related to the policy, practice, and organizational limits involved in the implementation of a program.
7	Wilson, McCormack (2006) Australia Nursing^(^ 18 ^)^	It is strongly linked to the Emancipatory Practice Development programs, supporting effective research questions that will test research outcomes and inform the possibility of transferring mechanisms in different contexts. The result depends on the context as it interferes with the mechanisms. It is based on the principles of realism and seeks to apprehend what is true (mechanisms that may or may not trigger), real (events that may or may not be observable but that exist), and empirical (evidence of experiences and observations made). The explanations mainly require interpretations of qualitative data to discover the reasoning and circumstances of the actors in specific contexts, not in their abstraction, which necessarily involves the participation of stakeholders and the identification of the local history.
8	Westhorp (2008) United Kingdom Philosophy^(^ 19 ^)^	It requires the theories and/or assumptions implicit in a program to be made explicit in order to determine what and how to assess. It implies identifying theoretical assumptions, resources, and activities of the program, related to the mechanisms that lead to short, medium, and long-term outcomes.
9	Keller, et al. (2009) Sweden Business^(^ 20 ^)^	It must be applied by those who wish to plan and implement innovations considering the receptive context for change, the readiness of the system for innovation, power relations, and the external socio-political context. After the innovation has been implemented, the realist evaluation can be applied to provide an explanation of the outcome patterns, depending on the mechanisms and on the contextual constraints.
10	Kontos, Poland (2009) Canada Rehabilitation^(^ 21 ^)^	The critical realist evaluation derives from critical realism, an approach that is advocated for implementing evidence-based innovation in health. The evaluation is inherent to the implementation process and not something out of place to measure outcomes. The context and the process are considered, that is, the conditions that promoted or hindered the changes. These can be assessed by combining quantitative and qualitative data, which will promote an understanding of why the intervention worked, for whom, and under which circumstances.
11	Coryn, Noakes, Westine (2011) USA Evaluation^(^ 22 ^)^	Five principles for a theory-oriented evaluation: 1) Theory-oriented evaluations/evaluators should create a plausible program theory; 2) Theory-oriented evaluations/evaluators should create and prioritize evaluation questions around a program theory; 3) The theory of the program should be used to guide the planning, design, and execution of the evaluation considering relevant contingencies; 4) Theory-oriented evaluations/evaluators must measure the constructs postulated in the program theory; 5) Theory-oriented evaluations/evaluators must identify breaks, side effects, determine the effectiveness (or ineffectiveness) of the program, and explain cause and effect associations between the theoretical constructs.
12	Westhorp, et al. (2011) Australia Did not describe^(^ 23 ^)^	It starts from the precept that social organization occurs in the form of systems. Social systems are open: the elements can enter and leave the system. As a result, any event has many causes and, at the same time, can have many consequences. It also means that every result of a program is the result of multiple causes. The findings are likely to focus on a subset of mechanism-contexts-outcomes. In general, they can indicate that: • a specific intervention works separately; • it is implemented in different ways; • it is more effective with some groups than with others; • it will have a greater use in one location than in another; • it has intentional and unintentional consequences; • its effects are likely to last.
13	Pawson, Manzano-Santaella (2012) United Kingdom Sociology and Social Politics^(^ 24 ^)^	Guided by theory and capturing the outcomes of all the interventions, aiming to identify what works, for whom, in which circumstances, under what aspects, for how long, and why. The complex range of outcomes must be explained to verify the program's effectiveness. Another objective of the realist evaluation is to improve the programs, distinguishing the effectiveness of the implementation (effective or ineffective).
14	Luskin, Ho (2013) USA Did not describe^(^ 25 ^)^	Social improvement must be considered as the objective of the collective evaluations so that program developers, participants, policy makers, and the general public can make decisions about programs and policies.
15	Souza (2013) Singapore Education^(^ 26 ^)^	It is possible to consider a social program as the input that will reconfigure or differently activate the underlying causal mechanisms within pre-existing social structures to generate change or a different potential within the action context. An action context comprises aspects of structure, culture, agency, and relationships.
16	Manzano (2016) United Kingdom Did not describe^(^ 27 ^)^	The research process will begin by creating theories, which will be tested, refined and re-tested and, in this iterative process, the understanding of the real world is also refined.
17	Wong, et al. (2016) United Kingdom Primary Care^(^ 28 ^)^	It must explain the underlying theories of a program, developing clear hypotheses about how and for whom the programs can work. It implies collecting data, not only about the impacts of the program or about the program implementation processes, but also about specific aspects of the context that can affect the intended and unintended outcomes of the program and about the specific mechanisms that may be creating changes. The evaluation must be properly described: what it consists of, who the target is, who provides it, what the geographic reach is, what is expected to be achieved, and so on. The data collected must include information about program impacts and implementation processes, specific aspects of the program context that can affect the program outcomes and how those contexts shape the specific mechanisms that may be creating changes. When seeking information from the participants, it is assumed that different participants have different perspectives, information, and understandings about how programs should work and whether they actually work. The realistic methodology is well suited to the study of Community-Based Participatory Research. General and specific limitations of the realist evaluations must be explained so that the readers can interpret the findings based on them. Points or limitations imposed by any changes made to the evaluation processes must also be reported and described.
18	Wong, et al. (2017) United Kingdom Primary Care^(^ 29 ^)^	It collects specific aspects of the context that may impact on the intended and unintended outcomes of the program, and the specific mechanisms that may be changing the outcomes.
19	Wong (2018) United Kingdom Primary Care^(^ 30 ^)^	It is initiated by the construction of a theory, that is, an explanation of how, why, for whom, in which contexts, and on which basis an intervention is designed to "work". The result of any phenomenon is derived from the context and from the (C+M=O) mechanism. Realist evaluations are research methodologies that explicitly and consistently link the context to the outcomes and set out to address complexity issues.

It was identified that the concepts presented by the different authors are congruent with each other. The realist evaluation is a methodology structured according to the philosophical assumptions of realism, which aims to address the complexity of the health interventions, considering the influence of the social relationships and organizational structures. It is a theory-oriented approach that focuses the evaluation on obtaining answers about what works, for whom, in which context, and how^(^ 6 ^,^ 8 ^,^ 16 ^,^ 29 ^-^ 30 ^)^.

The realist evaluation considers the Context-Mechanisms-Outcomes (CMO) articulation to understand the underlying dispositions that make up the studied situation^(^ 6 ^,^ 15 ^)^. The mechanisms are the way the subjects interpret and act regarding the intervention and are not always explicit, whereas the context is represented by the characteristics of the conditions in which the interventions were introduced. Finally, the outcomes are the consequences of activating different mechanisms in different contexts^(^ 14 ^,^ 17 ^)^, which produce clear theories or structured theoretical models, to explain how interventions promote the expected outcomes^(^ 22 ^)^.

The CMO configuration has the potential to introduce a broad and complete picture of what is happening, in order to elucidate the essential elements that enabled the use, or not, of interventions, programs, and/or public policies^(^ 13 ^)^, assuming that different contexts produce different outcomes, either better or worse^(^ 15 ^-^ 16 ^)^. 

Another innovative aspect is the purpose of understanding the configuration of outcome patterns obtained by implementing the interventions. The realist evaluation studies aimed to present the changes resulting from the implementation of interventions and how such measures were produced and introduced to modify the context and balance of the underlying mechanisms^(^ 15 ^,^ 20 ^)^.

In order to understand the investigated reality, the realist evaluation must begin with theories, which will be tested and refined in a cyclical and iterative way, being structured in the form of proposals about how the mechanisms occurred in contexts to produce outcomes^(^ 6 ^,^ 17 ^-^ 19 ^,^ 21 ^,^ 27 ^)^. In this sense, programs/interventions/policies are evaluated based on the changes produced in the individuals, subgroups, and contexts involved, in addition to identifying the social and cultural resources that are necessary to sustain the changes^(^ 6 ^)^. For the realist evaluation, it is necessary to: (1) formulate a theory about the program, interventions, and/or policies assessed; (2) formulate and prioritize evaluation questions around the theory; (3) plan, design, and conduct the evaluation based on the theory; (4) identify the constructs postulated in the theory; (5) determine the efficacy or effectiveness of the program, interventions, and/or policies assessed, and explain cause and effect associations between the theoretical constructs and the factors that affected the outcome pattern. These principles are situational and do not constitute strict criteria, since their application depends on the nature of the program assessed, the objective of the evaluation and the individuals who will use it^(^ 22 ^)^.

In this way, the evaluation allows for a circuit that can be guided by different strategies for understanding the reality under study^(^ 6 ^,^ 12 ^,^ 19 ^,^ 21 ^)^. The choice of the data collection methods must be guided by the theory, in order to test the assumptions/theories and to unveil the patterns and regularities of the program, through observations, data collection, data analysis, among others^(^ 6 ^,^ 27 ^)^. In complex programs, random sampling, randomized clinical trials or quasi-experimental projects may not be able to identify elements that interfere with the participation of those involved in the program, because the intervention has unexpected processes that cannot be predicted in advance for statistical purposes and do not capture outcomes and contextual elements^(^ 20 ^,^ 27 ^)^.

In this sense, it is necessary to include the evaluator in the process, who has the task of understanding and testing the theory studied^(^ 16 ^,^ 19 ^)^. This process should follow an emancipatory perspective, which requires the participation of other stakeholders in the evaluation, in the identification of local history, and in the transformative actions in which the practice occurs^(^ 18 ^)^. Emancipation is in this sense understood by the author as a process in which those involved identify needs for changes in the practices, reflect on such practices, and seek to promote cultural changes, based on the needs identified in that context. Analyses and changes are related to the local cultural dimension of the social relationships^(^ 18 ^)^. The political and economic dimensions are not considered in the author’s perspective.

The emerging realist evaluation is one of the evaluation categories found and is described as the one that aims at social improvement by implementing programs and policies. The evaluator engages in the process of creating knowledge with the participants, believing that public discussion informs society for making socially responsible decisions. Therefore, the evaluation is not a condition for deciding on the merit of a social policy, but it supports democratic decision-making processes^(^ 14 ^,^ 25 ^)^.

The critical realist evaluation derives from critical realism, an approach that is advocated for implementing evidence-based innovation in health. In this perspective, it is highlighted that the evaluation is inherent to the implementation process and not something out of place to measure outcomes. The critical realist evaluation emerges more explicitly to show that it is part of the broader implementation process considering the conditions that promoted or hindered the changes^(^ 21 ^)^.

In short, the realist evaluation has the potential to support decision-making and the creation of public policies, as the outcomes are presented according to the different contextual realities of pre-existing social structures, to generate a change that includes structures, culture, and social relationships^(^ 13 ^,^ 15 ^,^ 26 ^)^.

The integration of the stages was possible without the need to overcome contradictions, since all the papers found are based on the same epistemological and theoretical-methodological frameworks, with some non-conflicting variations, as is the case with the emerging and critical realist evaluation. Thus, from the CMO configuration, the stages for conducting the realist evaluation were integrated and shown in Figure 4.

**Figure 4:** Development stages of the realist evaluation. São Paulo, 2019

1. Definition of the theory and of the evaluation questions^(^ 17 ^,^ 23 ^)^
2. Title^(^ 28 ^)^
3. Abstract^(^ 28 ^)^
*Introduction* ^(^ 28 ^)^
4. Presentation of the study theme^(^ 28 ^)^
5. Theory of the program^(^ 28 ^)^
6. Evaluation questions, objectives, and focus^(^ 28 ^)^
*Methods*
7. Justification for using the realist evaluation^(^ 28 ^)^
8. Description of the evaluated program policy, initiative, or product^(^ 28 ^)^
9. Evaluation locus^(^ 28 ^)^
10. Description and justification of the evaluation design^(^ 28 ^)^
11. Data collection methods^(^ 17 ^,^ 23 ^,^ 27 ^-^ 28 ^)^
12. Definition of the participants^(^ 17 ^,^ 23 ^,^ 27 ^-^ 28 ^)^
13. Data analysis^(^ 17 ^,^ 27 ^-^ 28 ^)^
14. Ethical approval^(^ 28 ^)^
*Results* ^(^ 17 ^,^ 28 ^)^
15. Details of the participants^(^ 28 ^)^
16. Main results^(^ 28 ^)^
17. Theory Test^(^ 17 ^)^
*Discussion and final considerations* ^(^ 28 ^)^
18. Summary of the findings^(^ 28 ^)^
19. Strengths, limitations, and future directions^(^ 28 ^)^
20. Discussion and main conclusions^(^ 28 ^)^
21. Funding and conflict of interests^(^ 28 ^)^

Using the studies included and the analysis category, the specific guidelines for each stage were described, considering that the stages can occur in an overlapping and iterative way.

*1. Definition of the theory and of the evaluation questions:* Formulate theories about the program (what works, for whom, and in which context)^(^ 17 ^)^. The understanding of the theory allows for the development of experimental configurations of mechanisms and contexts. Consider that the explanation of reality by the theory will occur in all the stages of the evaluation^(^ 23 ^)^;

*2. Title:* Identify the document as a realistic synthesis or evaluation^(^ 28 ^)^;

*3. Abstract:* Include brief details about: the policy, program or initiative under evaluation; program configuration; purpose of the evaluation; evaluation question(s) and/or objective(s); strategy; data collection, documentation, and analysis methods; main findings and conclusions^(^ 28 ^)^;

*Introduction:*

*4. Presentation of the study theme:* Explain the purpose of the evaluation with secondary data^(^ 28 ^)^;

*5. Theory of the program:* Describe the initial program theory (or theories) that underpin the program, policy, or initiative^(^ 28 ^)^;

*6. Evaluation questions, objectives, and focus:* Indicate the question(s) and specify the objectives for the evaluation. Describe whether and how the program theory was used to define the scope and focus of the evaluation^(^ 28 ^)^;

*Methods:*

*7. Justification for using the realist evaluation:* Explain why a realist evaluation approach was chosen and (if relevant) adapted^(^ 28 ^)^;

*8. Description of the program policy, initiative or product assessed:* Provide relevant details about the program, policy or initiative assessed^(^ 28 ^)^;

*9. Evaluation locus:* Describe and justify the reason for choosing the place where the evaluation took place^(^ 28 ^)^;

*10. Description and justification of the evaluation design:* A description and justification of the evaluation design (i.e., the account of what was planned, done, and why) must be included, at least briefly or as an appendix, in the document that presents the main conclusions. If this does not happen, the omission must be justified and a reference or link to the design must be provided. It can also be useful to publish or avail for free (for example, online on a website) any document or original evaluation design document, if any^(^ 28 ^)^;

*11. Data collection method:* Provide details and justifications about the method choices, which can be quantitative or qualitative: which ones were used, why, and how they contributed to develop, support, refute or refine the program theory. Quantitative methods are mostly used regarding the context, for example, group comparison; Qualitative methods contribute to the exploration of hypotheses and to the identification of unforeseen elements of the context and outcome. The semi-structured qualitative interview is the most common and available method of data collection, either alone or in combination with other methods. It usually contains exploratory questions based on the program assessed and acting as instruments to extract the proposals of the general investigation^(^ 17 ^,^ 23 ^,^ 27 ^-^ 28 ^)^;

*12. Definition of the participants:* Describe how the participants in the evaluation were defined, invited and engaged, and how they contributed to the development, support, refutation or refinement of the program theory^(^ 28 ^)^. The stakeholders are considered the main data sources, as they have experience in specific phases and processes within the program assessed^(^ 17 ^)^;

*13. Data analysis:* Describe and justify which method of analysis was used, how the program theory was developed, supported, refuted and refined, and whether the analysis changed during the evaluation^(^ 28 ^)^. There is not just one suitable analytical method, as it depends on the theories proposed and on the availability of data^(^ 28 ^)^. In the realistic program theory, different outcome patterns are expected to exist for different groups or contexts within the program, and the analysis tests these theories^(^ 23 ^)^;

*14. Ethical approval:* Indicate whether the realist evaluation required and obtained ethical approval from the relevant authorities, providing details as appropriate. Explain the reason if it is not necessary to conduct the evaluation^(^ 28 ^)^.

*Results:*

*15. Details of the participants:* Report (if applicable) who participated in the evaluation, details of the data they provided, and how the data was used to develop, support, refute or refine the program theory^(^ 28 ^)^;

*16. Main results:* Present the main results, linking them to the contexts, mechanisms, and configurations of outcomes. Show how they were used to develop, test, or refine the program theory^(^ 28 ^)^;

*Discussion and final considerations:*

*17. Theory test:* Review and understand the possible CMO pattern configurations to refine the theory and promote knowledge^(^ 17 ^)^;

*18. Summary of the outcomes:* Summarize the main outcomes with attention to the evaluation questions, purpose, the program theory, and the stakeholders^(^ 28 ^)^;

*19. Strengths, limitations, and future directions:* Discuss the strengths of the evaluation and its limitations, including considerations of all the stages in the evaluation processes. In many evaluations, there will be an expectation of providing guidance on future directions for the program, policy or initiative, its implementation and/or project. The particular implications of the realistic nature of the outcomes must be reflected in these discussions. Consider that future directions support public policies, being important political instruments for social research^(^ 28 ^)^;

*20. Discussion and main conclusions:* Compare the outcomes with existing literature and list the main conclusions that are justified by the data analysis. If appropriate, offer recommendations consistent with a realistic approach^(^ 28 ^)^;

*21. Funding and conflict of interests:* Indicate the source of funding (if any) for the evaluation, the role played by the funder (if any), and any conflicts of interest of the evaluators^(^ 28 ^)^.

The aforementioned stages, integrated from the studies gathered in this review, allow guiding the development, as well as the preparation, of the final report of a realist evaluation^(^ 17 ^,^ 23 ^,^ 28 ^)^.

## Discussion

It was assumed in this work that the potential of the realist evaluation lies in its ability to capture the interactions inherent to the CMO complex.

This type of evaluation highlights that the outcomes found in a given evaluation process are not linearly transferable to other realities, as they depend on the interaction of particular social processes^(^ 23 ^)^.

The interactions, as analyzed by the mediation category in this work, represent the articulation between the parts of a complex totality and, at the same time, the movement between the singularity and the totality, formed by socio-historical structures, constituted by interactions with dynamic and contradictory movements, and not only by the Cartesian sum of the parts^(^ 31 ^)^.

The mediation category carries both an ontological and a reflective dimension since it exists independently of the social subject, and can support individuals’ reflection processes about a certain reality, captured by its essence and not only in the appearance realm^(^ 32 ^)^.

Mediation constitutes the ontology of the social being that is based on the own movement of the categories of reality, and not on logical ideal concepts, being present in the sociability of the social being. Therefore, it is sustained in the perspective of the relationship between man and nature and, in this way, the transformation of nature by man (work) is a condition of human existence^(^ 31 ^)^. In the realist evaluation, part of the underlying mechanisms, which can interfere with the outcomes of a given project, depends on the reflection of this social being on the proposed intervention and on the reality in which it is inserted.

This field of mediations takes shape in the particularity in which the dialectic between the universal and the singular occurs. It is in this mediation field that the singular facts are related to the laws of universality, which is configured from the reality of the singular. The individual, being the smallest unit of the social totality, has infinite variations and, therefore, has great complexity and particularities^(^ 31 ^)^.

The particular represents the expression of the categories of mediation between the singular individuals and society^(^ 31 ^)^. In the realist evaluation, the context is located in the particular dimension since, depending on its conditions, there will be or there will not be the activation of underlying mechanisms, which may influence the outcomes of the intervention proposed by a given program, policy or service.

It starts with the understanding that the social being and its dynamic complexes express themselves in a particular way. In the sphere of universality, there are the great determinations and tendency laws of a given social complex. Laws and determinations that, in the sphere of singularity, are hidden by the dynamics of facts^(^ 31 ^)^. Thus, the mediation category helps to apprehend the movement of the hidden social being in individuals and, therefore, supports the understanding of the underlying mechanisms addressed in the realist evaluation.

In this perspective, mediations are instrumental categories by which the action is implemented, they are a way of objectifying the practice. The mediations are expressed by the set of instruments, resources, techniques, and strategies that the subject becomes aware of in order to penetrate the plots of reality as a possibility to transform it^(^ 32 ^)^.

Regarding the limitations, realist evaluations of property demarcation and education programs in penitentiaries, initially described by the creators of the realist evaluation, were not based on theoretical frameworks or totalizing theories that explained what the root is of the problems that lead to the creation of these programs. Nor was it discussed, for example, how society is organized and the mode of capitalist production, which are generators of social inequalities. Thus, in its origin, the realist evaluation showed certain pragmatism when looking for cause and effect relationships^(^ 6 ^)^.

Other limitations concern the philosophical dimension of critical realism, which emphasizes that the production of several authors who follow the critical realistic theory is restricted to non-historical abstraction by not using any tools that make it possible to understand how objective social structures can be transformed and how to carry out the transformation. Thus, it states that critical theorists do not always make assumptions about the object of investigation when analyzing social relationships, which makes the starting point of investigation arbitrary. In this perspective, the causal mechanisms are self-referenced and the structures only exist as a result of human behavior and that the causal powers would thus be relational^(^ 33 ^)^.

Such limitations are still under discussion, even by one of the main theorists of critical realism - Roy Baskar - who has sought to add the dimensions of historicity and totality, and the dialectic, considering the contradictions of reality^(^ 34 ^)^. It has been argued that the use of dialectic alters the research strategy for critical realism in several ways. First, it demonstrates the need to abstract causal powers through dialectical connections and contradictions that are inherent to diverse interconnected totalities. Second, historical analysis becomes a key moment in this dialectical procedure in order to overcome a common dualism in critical realistic thinking between structures in closed systems and contingent mechanisms in contingent historical events. Society is seen as an interconnected historical totality, so that concrete events are themselves moments of that totality. Thus, the mechanisms are considered moments of dialectical connections in totalities and moments of specific dialectical contradictions. It is verified that the dialectic reshapes the debates about the relationship between the parts and the whole.

## Conclusion

This IR made it possible to map the scientific production of the health area on the concepts, purposes, and stages of the realist evaluation. The studies included did not present theoretical discrepancies, allowing for the epistemological reconciliation of the concepts and methodology of this approach. The integration of data enabled the presentation of 21 stages for the development of the realist evaluation of complex health contexts.

Thus, it can be said that, by analyzing the outcomes of a given intervention, based on the understanding of the interactions that occur in the reality of social relationships, the realist evaluation ends up identifying its potential for transformation and the elements that interfere in the outcome of the interventions.

From the milestones of the mediation category, however, there is a need for these theories to be elaborated incorporating the elements of the social macrostructure to which the mechanisms are connected.

For collective health, this has essential implications for research and health policies because taking social totality as a reference expands the explanation about reality and, consequently, the possibilities for transformation.

## References

[1] Craig P, Dieppe P, Macintyre S, Michie S, Nazareth I, Petticrew M (2013). Developing and evaluating complex interventions: the new medical
research council guidance. Int J Nurs Stud.

[2] World Health Organization (2013). Evaluation practice handbook.

[3] Moore GF, Audrey S, Barker M, Bond L, Bonell C, Hardeman W (2015). Process evaluation of complex interventions: Medical Research
Council guidance. BMJ.

[4] Fowler Davis S, Hinde S, Ariss S (2020). Complex programme evaluation of a "new care model" vanguard: a
shared commitment to quality improvement in an integrated health and care
context. BMJ Open.

[5] Tinoco DS, Souza LM, Oliveira AB (2011). Public policies evaluation: traditional and pluralista
models. Rev Pol Públ.

[6] Pawson R, Tilley N (1997). Realistic Evaluation.

[7] Yonekura T, Quintans JR, Soares CB, Negri AAD (2019). Realist review as a methodology for using evidence in health
policies: an integrative review. Rev Esc Enferm USP.

[8] Marchal B, van Belle S, van Olmen J, Hoeree T (2012). Is realist evaluation keeping its promise: A review of published
empirical studies in the field of health system research. Evaluation.

[9] Whittemore R, Knafl K (2005). The integrative review: updated methodology. J Adv Nurs.

[10] Soares CB, Hoga LAK, Peduzzi M, Sangaleti C, Yonekura T, Silva DRAD (2014). Integrative review: concepts and methods used in
nursing. Rev Esc Enferm USP.

[11] Soares CB, Campos CMS, Yonekura T (2013). Marxism as a theoretical and methodological framework in
collective health: implications for systematic review and synthesis of
evidence. Rev Esc Enferm USP.

[12] Moher D1, Liberati A, Tetzlaff J, Altman DG, PRISMA Group (2009). Preferred reporting items for systematic reviews and
meta-analyses: the PRISMA statement. PLoS Med.

[13] Henry GT, Rog DJ, Henry GT, Julnes G, Mark MM (1998). A realist theory and analysis of utilization. Realist evaluation: an emerging theory in support of practice: new
directions for evaluation.

[14] Mark MM, Henry GT, Julnes G, Henry GT, Julnes G, Mark MM (1998). A realist theory of evaluation practice. Realist evaluation: an emerging theory in support of practice.

[15] Tilley N (2000). Realistic evaluation: an overview.

[16] Kazi MAF, Rostila I (2002). The practice of realist evaluation in two countries.

[17] Pawson R, Tilley N (2004). Realist evaluation.

[18] Wilson V, McCormack B (2006). Critical realism as emancipatory action: the case for realistic
evaluation in practice development. Nurs Philos.

[19] Westhorp G (2008). Development of realist evaluation models and methods for use in
small-scale community based settings..

[20] Keller C, Gäre K, Edenius M, Lindblad S (2009). Designing for complex innovations in health care: design theory and
realist evaluation combined.

[21] Kontos PC, Poland BD (2009). Mapping new theoretical and methodological terrain for knowledge
translation: contributions from critical realism and the
arts. Implement Sci.

[22] Coryn CLS, Noakes LA, Westine CD (2010). A systematic review of theory-driven evaluation practice from
1990 to 2009. Am J Eval.

[23] Westhorp G, Prins E, Kusters CSL, Hultink M, Guijt IM, Brouwers JHAM (2011). Realist Evaluation: an overview.

[24] Pawson R, Manzano-Santaella A (2012). A realist diagnostic workshop. Evaluation.

[25] Luskin RJ, Ho T (2013). Comparing the intended consequences of three theories of
evaluation. Eval Program Plann.

[26] ouza DE (2013). Elaborating the Context-Mechanism-Outcome configuration (CMOc) in
realist evaluation: a critical realist perspective. Evaluation.

[27] Manzano A (2016). The craft of interviewing in realist evaluation. Evaluation.

[28] Wong G, Westhorp G, Manzano A, Greenhalgh J, Jagosh J, Greenhalgh T (2016). RAMESES II reporting standards for realist
evaluations. BMC Med.

[29] Wong G, Westhorp G, Greenhalgh J, Manzano A, Jagosh J, Greenhalgh T (2017). Quality and reporting standards, resources, training materials and
information for realist evaluation: the RAMESES II project..

[30] Wong G (2018). Getting to grips with context and complexity - the case for
realist approaches. Gac Sanit.

[31] Pontes RN (1990). A Propósito da Categoria de Mediação. Serviço Social e Sociedade.

[32] Martinelli ML (1993). Notas sobre mediações: alguns elementos para sistematização da
reflexão sobre o tema. Serv Soc Soc.

[33] Roberts JM (2012). Marxism and Critical Realism: The Same, Similar, or Just Plain
Different?. Cap Cl.

[34] Roberts JM (2014). Critical Realism, Dialectics, and Qualitative Research
Methods. J Theory Soc Behav.

